# Cross-Contamination Identification of Additive Manufacturing Metal Powders Using Spatially Confined Particle-Flow LIBS and Machine Learning

**DOI:** 10.3390/s26123591

**Published:** 2026-06-06

**Authors:** Leiyi Ding, Dan Feng, Yinghao Wang, Mengjie Shan, Yuanbin Wang, Nan Ma

**Affiliations:** 1 Analytical & Testing Center, School of Mechanical Engineering, Northwestern Polytechnical University, Xi’an 710072, China; 2 Shenzhen Research Institute, Northwestern Polytechnical University, Shenzhen 518057, China

**Keywords:** laser-induced breakdown spectroscopy (LIBS), additive manufacturing, metal powders, cross-contamination, machine learning, online quality monitoring

## Abstract

Laser-induced breakdown spectroscopy (LIBS) offers rapid, in situ, and multi-element detection, and therefore shows strong potential for quality monitoring of metal powders in additive manufacturing. However, direct LIBS analysis of flowing metal powders is often affected by particle splashing, unstable laser–particle coupling, and plasma fluctuations, which reduce signal repeatability and detection reliability. To address these issues, this study developed an integrated measurement and classification framework for identifying cross-contamination in additive-manufacturing metal powders. A stable powder particle stream was generated through vibratory feeding and particle-flow focusing, while a hollow quartz tube with a side opening was introduced to provide cylindrical spatial confinement, thereby improving the stability of laser–particle interaction and enabling in situ spectral acquisition without pellet preparation. TC4 powder was used as the base material and AlSi10Mg powder as the contaminant, and samples with contamination levels of 0, 0.5, 1, 2, and 5 wt.% were prepared. Two independent batches of single-shot LIBS spectra were collected. To reduce the influence of strong spectral fluctuations, outlier spectra were removed using full-spectrum total-intensity quantile filtering, followed by asymmetric least-squares baseline correction and standard normal variate transformation. PCA combined with multiple machine-learning models was then applied for contamination identification. The results showed that LIBS spectra at different contamination levels exhibited distinguishable distributions in principal-component space, and the spectral differences between clean and contaminated powders became more pronounced with increasing contamination level. In binary classification, several models achieved high classification accuracy at medium and high contamination levels, while PCA-SVM-RBF showed the best performance at low concentrations. In five-class cross-validation, the 5 wt.% class exhibited the clearest decision boundary, whereas confusion remained among low and adjacent contamination levels, indicating that contamination-induced spectral responses followed a more continuous transition. These results demonstrate that the proposed spatially confined particle-flow LIBS framework combined with machine-learning classification can effectively achieve rapid identification of cross-contamination in additive-manufacturing metal powders and provides a feasible technical route for online powder quality monitoring.

## 1. Introduction

Additive manufacturing (AM) is reshaping multiple sectors of the aerospace industry by enabling the fabrication of geometrically complex, high-performance, and high-precision components while improving material utilization and reducing manufacturing cost. It has therefore played an increasingly important role in commercial and military aircraft, space applications, and missile systems [1,2,3]. In industrial practice, however, the layer-by-layer deposition nature of AM often requires substantial processing time, and once abnormal performance is identified after fabrication, reproducing the entire component is extremely costly [4]. Consequently, real-time detection of anomalies during the deposition process is of great importance. Among the various factors affecting part quality, powder cross-contamination is widely recognized as a critical source of abnormal performance in laser-based additive manufacturing. Such contamination may occur at multiple stages of the process chain, including powder production, storage, and bulk-part fabrication [5,6]. In this context, material cross-contamination refers to the introduction of trace foreign materials into the feedstock powder owing to factors such as inadequate machine cleaning after previous builds or insufficient quality control during powder production and storage. Even minor contamination can induce detrimental microstructural changes in AM components and thereby degrade their functional properties. To detect such contamination in a timely manner, several approaches have been explored. Mohammad Montazeri et al. [7] developed and applied a spectrogram-theory-based method to identify material cross-contamination in real time using in situ sensor signals acquired during fabrication. Marta Ceroni et al. [8] proposed a multivariate quantitative method based on ultraviolet–visible–near-infrared spectroscopy, which was able to detect contamination levels as low as 0.5 vol.% and identify both contaminant type and concentration. Eleonora Santecchia et al. [9] used scanning electron microscopy (SEM) and energy-dispersive spectroscopy (EDS) to identify and quantify cross-contamination in Ti-6Al-4V and maraging steel powders, thereby providing a basis for further applications in machine-learning-assisted analysis. Gowtham et al. [10] showed that recycled powders may contain process-affected particles with physical and chemical properties different from those of virgin powders, highlighting the importance of powder quality inspection prior to reuse. Overall, rapid and timely monitoring of powder quality during laser additive manufacturing remains highly necessary, while related studies are still limited and the associated technologies remain at an exploratory stage. Therefore, this study proposes a spatially confined particle-flow LIBS integrated measurement and classification framework for identifying cross-contamination in metal powders for additive manufacturing.

Laser-induced breakdown spectroscopy (LIBS) uses a focused high-energy pulsed laser to ablate a small amount of material from the sample surface, generating a localized plasma plume. By collecting and analyzing the characteristic emission lines of atoms and ions in the plasma, qualitative identification and quantitative determination of the chemical composition can be achieved based on spectral wavelength and intensity information. Owing to its broad elemental coverage, minimal sample preparation requirements, rapid response, suitability for real-time online detection, and good quantitative capability, LIBS has shown strong potential for in situ monitoring of powders in laser additive manufacturing [11,12,13].

Despite its broad applicability, direct LIBS analysis of powder samples remains challenging because the laser–material interaction can disturb and scatter loose particles. When the laser pulse interacts with the material, shock waves and mechanical disturbances are generated, which can cause particle fragmentation and powder splashing, thereby affecting the stability of the plasma morphology. Previous studies on LIBS detection under different additive-manufacturing scenarios have consistently reported poor repeatability when metal powder jets are analyzed directly [14,15,16]. To address this issue, Suyanto et al. [17] developed a technique to bend and confine the plasma within a sample holder and then detect plasma emission from the shockwave-bent plasma outside the holder for powder analysis. Their results demonstrated a practical approach for handling small sample quantities and low background emission; however, the requirement for low-pressure conditions limits its applicability to in situ analysis. Guo et al. [18] from our group showed, using Fe-Mn mixtures, that compared with the pellet method, the double-sided tape method could significantly reduce prediction error. However, this approach still requires sample pretreatment and preparation. It has also been reported that double-pulse LIBS systems can be used for direct powder analysis [19], in which two lasers with different wavelengths are employed to control dust generation under low-pressure conditions.

To overcome the problems of powder splashing, plasma instability, and insufficient repeatability in direct LIBS detection of flowing metal powders, this study developed an integrated measurement and identification framework that combines stable particle-flow transport, spatial confinement enhancement, single-shot spectral quality screening, and machine-learning-based classification. Based on this framework, the spectral response characteristics and classification performance under different contamination levels were systematically evaluated, and the generalization capability of the method was further validated using an independent external test set. The proposed approach provides a feasible technical route for rapid identification of cross-contamination in metal powders for additive manufacturing.

## 2. Materials and Methods

### 2.1. Experimental Setup

**Figure 1:**
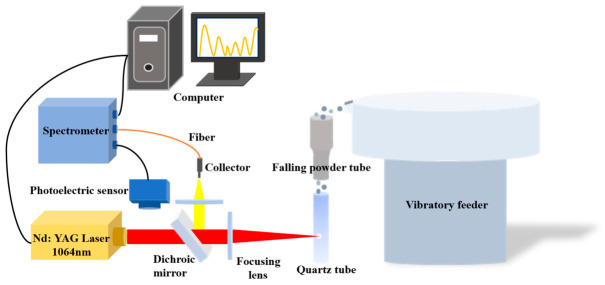
Schematic diagram of the spatially confined particle-flow LIBS experimental setup for cross-contamination identification.

As shown in Figure 1, the experimental setup consisted of a laboratory-developed miniaturized LIBS detection unit, a piezoelectric vibratory feeder, a particle-flow focusing device, and a cylindrical spatial confinement structure. TC4 powder was selected as the base material, while AlSi10Mg powder was used to simulate cross-contamination that may occur during the manufacturing process. The piezoelectric vibratory feeder delivered the powder into the transport channel through vibration, forming a particle stream with a stable flow velocity, uniform mass flow, and an approximate diameter of 4 mm. A quartz tube with an inner diameter of 4 mm and an outer diameter of 6 mm was installed at the lower end of the particle stream as the confinement structure. A circular hole with a diameter of 1.5 mm was opened on the side wall of the quartz tube to serve as the laser incident window, with the center of the hole located approximately 6 mm from the bottom end of the tube. The powder flows through the central region of the quartz tube as a free-falling, continuous stream of particles without causing blockage. Furthermore, the operating parameters of the vibratory feeder, including vibration frequency and amplitude, were adjusted using the feeder controller. The resulting powder mass flow rate was calibrated by measuring the time required for 100 g of powder to pass through the feeding system. Based on repeated measurements, the average mass flow rate of the particle stream was maintained at approximately 0.5 ± 0.08 g/s.

In the miniaturized LIBS system, a RealLight passive Q-switched pulsed laser (PQE-1064-0.01-10) was used as the excitation source. The emitted 1064 nm pulsed laser had a pulse energy of approximately 24 mJ, a repetition rate of 10 Hz, and a pulse duration of approximately 10 ns. The incident laser beam diameter was approximately 4 mm, and the beam was focused into the particle-flow region using a lens with a focal length of 15 mm. Based on the diffraction-limited estimation, the nominal focused spot diameter was approximately 5 μm. The laser beam was delivered through the terminal optical path to interact with the particle stream and generate plasma. The plasma emission was collected by an optical probe and coupled into an AVANTES single-channel ultraviolet miniature spectrometer (AvaSpec-Mini4096CL) for spectral acquisition in the 200–400 nm range. To achieve synchronized triggering, an LBT EK InGaAs fixed-gain amplified photodetector (IAF-800B050, sensitive at 1064 nm) was positioned at the laser exit. The electrical pulse output from the photodetector was used as the external trigger signal for the spectrometer, thereby enabling the setting of acquisition delay and stable synchronization of spectral collection.

### 2.2. Particle-Flow LIBS Spectral Acquisition Under Spatial Confinement

To improve LIBS signal quality, spatial confinement was introduced as an enhancement strategy. Spatial confinement improves plasma emission by restricting the free expansion of the laser-induced plasma with a physical barrier. The underlying mechanism is that, when laser breakdown generates plasma, the accompanying shock wave is reflected by the confinement structure. The reflected shock wave then causes secondary compression and reheating of the plasma and regulates its expansion morphology and spatial distribution, thereby enhancing emission intensity and improving signal repeatability [20].

Based on our previous studies on spatial confinement structures for gas–solid two-phase systems [21,22,23], a hollow quartz tube with an outer diameter of 6 mm and an inner diameter of 4 mm was designed and fabricated to form a cylindrical confinement structure. To avoid laser ablation of the quartz wall, a side opening was machined on the tube wall so that the laser could directly pass through the opening and interact with the particle stream, while ensuring that the confinement structure did not interfere with the incident laser path or beam propagation.

For powder preparation, Ti-6Al-4V (TC4) powder was used as the base material and AlSi10Mg powder was used as the contaminant. Both powders had a particle-size range of 15–53 μm. Powder samples with contamination levels of 0, 0.5, 1, 2, and 5 wt.% were prepared. For each contamination level, two independent batches were produced. The corresponding masses of TC4 and AlSi10Mg powders were first weighed using an analytical balance and manually premixed to achieve preliminary homogenization. The premixed powders were then homogenized in a planetary ball mill at 400 rpm for 2 h with a ball-to-powder ratio (BPR) of 15:1. A stainless-steel milling jar and 10 mm stainless-steel balls were used, and the milling process was conducted in a sealed condition to reduce environmental exposure. After milling, the powders were recovered and sieved through a 53 μm mesh to break up soft agglomerates and improve the consistency of powder handling and subsequent LIBS measurements. All prepared powder batches were then sealed and stored for later testing.

LIBS spectra were collected for powders with different contamination levels. During acquisition, the metal powder was uniformly discharged from the vibratory feeder outlet and focused by the powder-fall tube into a particle stream with a diameter of approximately 4 mm. A stable measurement region of flowing metal particles was formed inside the cylindrical quartz tube. The laser passed through the opening in the cylindrical confinement structure and interacted with the flowing particles to generate the plasma plume collected by the spectrometer. The spectrometer integration time was set to 20 μs. For each contamination level, approximately 1000 single-shot LIBS spectra were collected from each of the two independent batches. One batch was used as the model-development dataset, and the other independent batch was reserved for external-test evaluation in the binary classification tasks. The detailed grouping strategy for binary and five-class classification is described in Section 2.4.

### 2.3. Preprocessing of Flowing Metal Powder LIBS Spectra

LIBS spectra are typically characterized by high complexity and redundancy. Appropriate data preprocessing can extract informative features from raw spectra and reduce the interference of redundant and irrelevant information, thereby improving the reliability of LIBS detection and analysis [24]. When LIBS is directly applied to gas–solid two-phase flow systems, the measurement process is easily affected by multiple unfavorable factors, resulting in large signal fluctuations, poor repeatability, and insufficient spectral representativeness, which ultimately reduces detection accuracy [25].

During in situ detection of flowing metal powders, particle position and density inevitably fluctuate during falling and transport. Such fluctuations directly affect the stability of laser–particle coupling and may cause focal-position deviation and reduced ablation efficiency [26]. In addition, the particle-size distribution and its uniformity also play an important role in ablation quality [27]. In practical flow conditions, part of the powder may fall in an agglomerated state, whereas other particles may be more uniformly dispersed. Variations in the effective ablated mass under different ablation conditions further influence plasma generation and evolution, leading to pronounced instability in both the intensity and morphology of the recorded spectra.

The raw LIBS data were acquired using the AvaSoft 8 software provided with the Avantes spectrometer and exported as wavelength–intensity matrices. Single-shot spectra were first subjected to quality screening. For each spectrum, the total spectral intensity over the full wavelength range was calculated, and outlier spectra with total intensities outside the batch-wise quantile thresholds were removed, specifically those below the 2nd percentile and above the 99.5th percentile. This step was used to exclude weak-breakdown or non-breakdown events, as well as abnormally strong breakdown events.

After spectral quality screening, each single-shot spectrum was processed using asymmetric least squares (ALS) baseline correction to suppress the continuous background emission. Negative residual intensities after baseline correction were truncated to zero. To further reduce shot-to-shot intensity variation, standard normal variate (SNV) transformation was applied to each spectrum by normalizing it with its own mean and standard deviation. The same preprocessing procedure was applied to spectra collected from all batches.

### 2.4. Metal Powder Cross-Contamination Identification Model

After adequate spectral preprocessing, different machine-learning models were employed for classification modeling and comparison. In practice, no single algorithm consistently performs best across all classification tasks [28]. Even relatively simple classification or chemometric methods may outperform more complex machine-learning approaches when appropriate data preprocessing is applied [29]. Therefore, five representative algorithms commonly used in spectral classification studies were selected in this work, namely LDA, PLS-DA, SVM-RBF, RF, and XGBoost. Among them, LDA represents a linear discriminant method, PLS-DA represents a latent-variable discriminant method suitable for high-dimensional collinear spectral data, and SVM-RBF represents a nonlinear classification method based on kernel mapping. RF and XGBoost represent two typical ensemble tree-based approaches based on bagging and boosting, respectively. RF mainly emphasizes variance reduction and robustness improvement, whereas XGBoost places greater emphasis on bias reduction and enhanced representation of complex feature relationships. By simultaneously introducing these models, the applicability of different modeling strategies to cross-contamination identification in metal powders could be systematically evaluated from the perspectives of linear discrimination, latent-variable extraction, kernel methods, and different ensemble mechanisms of tree-based models.

For the binary classification task, spectra from one independently prepared batch were used as the model-development dataset, within which ten-fold cross-validation was conducted for model training and evaluation. Spectra from the other independent batch were used exclusively as an external test set for final evaluation of model generalization ability. The contaminated powder class was defined as the positive class and the pure TC4 class as the negative class, so that Recall, F1, and AUC directly reflected the model’s ability to detect cross-contaminated samples. For the five-class classification task, stratified ten-fold cross-validation was conducted within the model-development batch to examine whether different contamination levels could be distinguished based on their LIBS spectral features. In each fold, approximately 90% of the spectra were used for training and the remaining 10% were used as the held-out fold, while the class distribution across the five contamination levels was preserved. For feature-processing steps such as standardization and PCA, fitting was performed only within each cross-validation training fold and then applied to the corresponding hold-out fold. In the external-test evaluation, standardization and PCA were fitted only on the model-development dataset and then applied to the independent external test set to avoid information leakage. For PCA-based models, the number of principal components was determined by retaining 95% of the cumulative explained variance. The hyperparameter settings of SVM-RBF, RF, and XGBoost are summarized in Table 1. All model settings were determined using the model-development dataset, and the independent external test set was used only for final evaluation. All algorithms were implemented in Python 3.8 for spectral preprocessing, model training, and performance evaluation.

## 3. Results and Discussion

### 3.1. Characteristic Spectral Line Analysis

**Figure 2:**
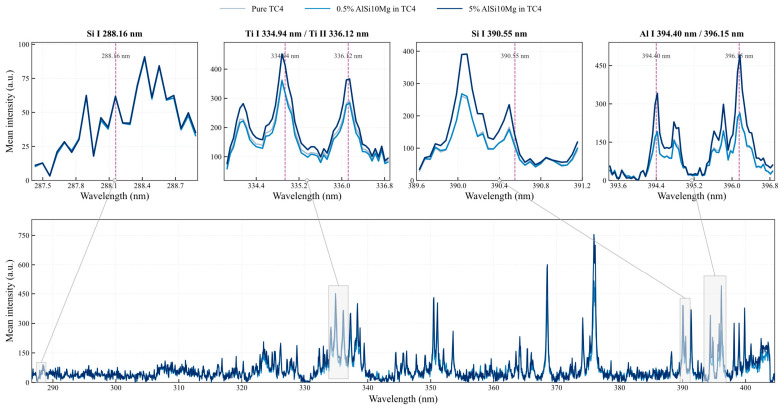
Full-spectrum LIBS profiles of TC4 powders with different AlSi10Mg contamination levels over the effective wavelength range. The inset plots show enlarged views of representative characteristic spectral regions, including Si I 288.16 nm, Ti I 334.94 nm/Ti II 336.12 nm, Si I 390.55 nm, and Al I 394.40 nm/396.15 nm.

The LIBS spectra acquired from pure TC4 powder and AlSi10Mg-contaminated TC4 powder exhibited clear multi-element emission characteristics. Each single-shot LIBS spectrum contained 2048 wavelength channels over the range of 286.64–404.63 nm, covering the near-ultraviolet to visible blue region. Figure 2 shows the plasma emission spectra of metal powders with different contamination levels. Overall, the spectral lines with relatively high analytical value and strong emission intensity were mainly concentrated in the regions of 287–288 nm, 318–338 nm, 363–376 nm, 389–396 nm, and 398–400 nm, whereas the other regions showed weaker emission intensity or fewer distinguishable features. Although Mg is an analytically relevant element for AlSi10Mg contamination, the Mg I 285.21 nm line was slightly outside the effective exported wavelength range used in this study (286.64–404.63 nm), and therefore it was not included in the present characteristic-line analysis.

Based on the relative line intensity, central wavelength, and elemental composition of the samples, and with reference to the NIST Atomic Spectra Database [30], the major emission features were assigned to Ti, Al, Si, and V. Among them, Si I 288.16 nm and Si I 390.55 nm were identified as the most important characteristic lines for recognizing AlSi10Mg contamination. Al I 394.40 nm and Al I 396.15 nm served as auxiliary discriminative lines. In contrast, Ti I/Ti II lines were densely distributed in the ranges of 334–338 nm, 363–376 nm, and 398–400 nm, forming the major spectral fingerprint of the TC4 matrix. In addition, several V I-related emission peaks could also be observed near 318 nm and 370 nm. Possible Fe contamination from the stainless-steel milling media was considered; however, no pronounced or isolated Fe-related peaks were observed within the effective spectral range, and weak responses near possible Fe lines overlapped with Ti- or Al/Si-related regions.

According to the above spectral assignment results, approximately 21 candidate atomic lines directly relevant to the classification task were preliminarily identified within the investigated wavelength range, corresponding to about 16–18 distinguishable characteristic peaks or peak clusters. The identification and labeling of these lines provide a basis for subsequent feature extraction, contamination discrimination, and classification model development. To illustrate the spectral variations more clearly, several representative atomic lines were selected for comparison among the 0 wt.%, 0.5 wt.%, and 5 wt.% samples, representing the clean, low-contamination, and high-contamination conditions, respectively, as shown in Figure 2.

Figure 2 provides an overall view of the LIBS spectral profiles of pure TC4 and AlSi10Mg-contaminated TC4 powders. The three spectra show similar global spectral patterns, indicating that the main spectral framework is still dominated by the TC4 matrix. However, noticeable intensity and peak-shape differences can be observed in several local wavelength regions after the introduction of AlSi10Mg. These differences are particularly evident in the enlarged inset regions, where Ti-related matrix emission bands and Al/Si-related characteristic features exhibit different responses to the contamination level. Therefore, the full-spectrum profile suggests that the spectral effect of AlSi10Mg contamination is not reflected by a single isolated emission line, but by combined variations across multiple characteristic regions.

As shown in the enlarged Al-related inset regions in Figure 2, the Al I 394.40 nm and Al I 396.15 nm lines exhibited clear response differences across contamination levels. Compared with pure TC4 powder, the introduction of AlSi10Mg led to an overall enhancement of the Al-related emission lines. In general, the 5 wt.% contaminated samples showed the highest peak intensity, followed by the 0.5 wt.% samples, indicating an increasing trend with increasing contamination level. This suggests that the Al characteristic lines are sensitive to cross-contamination and provide important discriminative information for distinguishing clean and contaminated powders. However, it should be noted that Al is present in both TC4 and AlSi10Mg. Therefore, the enhancement of Al emission lines should not be interpreted as direct evidence of contamination itself, but rather as an overall increase in the emission response of this shared element after the contaminant is introduced.

As shown in the enlarged Si-related inset regions in Figure 2, the Si-related emission lines also responded to AlSi10Mg contamination, although their variations were less stable than those of the Al lines. In some wavelength regions, the average peak intensity of the 5 wt.% contaminated sample was even lower than that of the 0.5 wt.% sample, indicating that the Si emission intensity did not increase monotonically with contamination level. An indicative LOD/LOQ estimation was therefore attempted for the Si I 288.16 nm and Si I 390.55 nm emission lines using the conventional 3σ/S and 10σ/S criteria. However, because the Si-line intensities did not show a monotonic or sufficiently linear response under the present particle-flow LIBS conditions, the resulting single-line LOD/LOQ values were not considered suitable for representing the detection capability of the proposed multivariate classification framework. This result indicates that single-line Si intensity is insufficient for robust contamination evaluation in flowing-powder LIBS and further supports the use of full-spectrum machine-learning classification in this study.

The Ti-related lines mainly reflect the matrix characteristics of TC4 powder. As the AlSi10Mg contamination level increased, the relative Ti content in the mixed system decreased because the contaminant powder does not contain Ti, which may lead to an overall weakening trend in the Ti characteristic lines. Therefore, identification of this type of cross-contamination should not rely on the appearance or variation of a single spectral peak alone, but rather on the overall spectral information.

### 3.2. Spectral Clustering Analysis

LIBS spectra are inherently high-dimensional and multivariate, containing not only informative features related to differences in elemental composition, but also random noise and redundant wavelength variables. Therefore, principal component analysis (PCA) was applied to reduce data dimensionality and to extract the dominant principal components that represent the major variations among samples.

**Figure 3:**
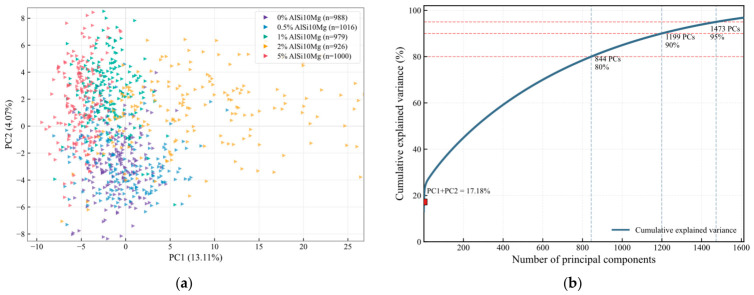
PCA of LIBS spectra from TC4 powders with different AlSi10Mg contamination levels. (**a**) PC1–PC2 score plot. (**b**) Cumulative explained variance as a function of the number of principal components.

As shown in Figure 3a, the first principal component (PC1) and second principal component (PC2) explained 13.11% and 4.07% of the spectral variance, respectively, giving a cumulative explained variance of 17.18%. The relatively low cumulative contribution of the first two principal components indicates that the discriminative information contained in the LIBS spectra was not concentrated along a single dominant direction, but rather distributed across multiple latent variables and wavelength dimensions. To further clarify the variance distribution of the high-dimensional LIBS spectra, the cumulative explained variance was calculated as a function of the number of principal components. As shown in Figure 3b, 844, 1199, and 1473 principal components were required to explain 80%, 90%, and 95% of the total variance, respectively. This result further confirms that the spectral variance of particle-flow LIBS data was distributed across a large number of latent variables rather than being concentrated in the first two principal components. Therefore, the PC1–PC2 score plot should be interpreted mainly as a low-dimensional visualization of the sample distribution, while subsequent PCA-based classification models retained components explaining 95% of the cumulative variance to preserve sufficient spectral information.

Nevertheless, the PC1–PC2 score plot still revealed a certain degree of separation among samples with different contamination levels. Pure TC4 samples were mainly distributed on one side of PC1, whereas samples contaminated with AlSi10Mg exhibited a systematic shift in principal-component space. In particular, a gradual trend along the PC2 direction was observed with increasing contamination level. These results indicate that variations in contamination level induced continuous changes in the overall spectral structure, thereby generating partially separable distribution patterns in the reduced-dimensional space. In other words, although PC1 and PC2 did not capture most of the total spectral variance, the spectral response differences among contamination levels were still sufficiently pronounced to be visualized by PCA, providing a basis for subsequent machine-learning classification.

However, PCA preprocessing was not equally suitable for all classification models. For PLS-DA, experimental results showed that introducing PCA before modeling led to performance deterioration. This may be because PLS-DA itself extracts latent variables in a supervised manner according to the class information, and additional PCA preprocessing may compress or discard low-variance but still discriminative information before model construction. In contrast, LDA is more sensitive to high-dimensional redundancy and collinearity in the input features, and its performance was substantially improved after PCA preprocessing. This improvement can be attributed to the ability of PCA to compress redundant information, alleviate collinearity, and stabilize the feature space. For SVM-RBF, which already has strong nonlinear modeling capability and good adaptability to high-dimensional data, the role of PCA was mainly to reduce noise and redundancy, and its effect on final performance was generally limited to small fluctuations. As for tree-based models such as RF and XGBoost, these methods can directly identify informative variables in the original high-dimensional feature space through feature selection and nonlinear splitting. Converting the original spectral variables into linear combinations by PCA may instead weaken the physical interpretability of local characteristic peaks and threshold-based splits. Therefore, PCA preprocessing was generally unnecessary for these models. Considering both the algorithmic characteristics and the experimental results, PCA preprocessing was finally applied to the LDA and SVM models, whereas PLS-DA, RF, and XGBoost were built directly on the full-spectrum data.

### 3.3. Machine-Learning Results for Cross-Contamination Identification

**Figure 4:**
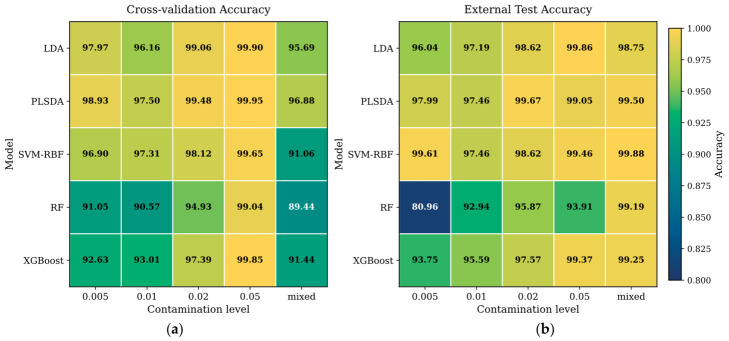
Heatmaps of classification accuracy of different machine learning models under different contamination levels: (**a**) cross-validation accuracy; (**b**) external test accuracy.

The identification performance of different models was evaluated under different contamination levels. Considering that the contamination level of polluted powders may be non-uniform in practical industrial scenarios, an additional binary classification task was constructed by randomly selecting 200 spectra from each contamination level and 800 spectra from the clean powder class. Figure 4a,b presents the cross-validation accuracy and external test accuracy of different machine-learning models under different contamination conditions.

Overall, the classification accuracy of all models showed an increasing trend as the contamination level increased from 0.5 wt.% to 5 wt.%, indicating that the LIBS spectral differences between contaminated powders and pure TC4 powder became progressively more pronounced with increasing contamination level. Under the medium-contamination (2 wt.%) and high-contamination (5 wt.%) conditions, most models achieved high classification accuracy in both cross-validation and external testing. In particular, PCA-LDA, PLS-DA, PCA-SVM-RBF, and XGBoost generally reached accuracies above 97%, while under the 5 wt.% and mixed-contamination conditions, some models further approached 99% accuracy. In general, the cross-validation and external test results exhibited similar trends, indicating that the proposed method has good generalization potential.

In addition, most models showed broadly consistent trends between the cross-validation and external test results, indicating that the developed models possessed good generalization ability rather than merely fitting the training data well. Overall, PCA-SVM-RBF achieved the best performance in the low-concentration identification task, while PCA-LDA, PLS-DA, and XGBoost also exhibited strong and stable classification capability. These results demonstrate that LIBS can not only effectively distinguish clean powders from contaminated powders, but that its identification performance further improves with increasing contamination level, thereby providing a reliable basis for subsequent low-concentration contamination detection and mixed-contamination screening.

For powder cross-contamination detection, the 0.5 wt.% condition represents the key scenario for distinguishing model performance. Under this low-concentration condition, PCA-SVM-RBF achieved the highest accuracy on the external test set (99.61%), clearly outperforming RF and XGBoost, which indicates its stronger nonlinear discriminative capability under weak spectral differences. PCA-LDA and PLS-DA also achieved relatively high external test accuracies (96.04%) at this low contamination level, suggesting that LIBS combined with appropriate machine-learning models can still enable effective identification even when contamination is limited. In contrast, RF achieved only 80.96% external test accuracy at 0.5 wt.%, and its performance at 5 wt.% was even lower than that at 2 wt.%, indicating comparatively weak robustness to low-concentration spectral fluctuations and batch-to-batch variation in the present dataset.

**Figure 5:**
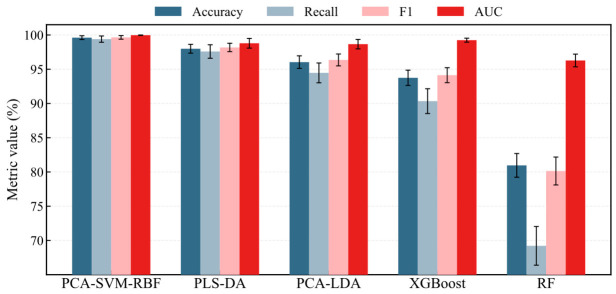
External-test classification performance of different machine-learning models under the 0.5 wt.% low-contamination condition. Error bars represent 95% confidence intervals estimated by bootstrap resampling of the external test set with 1000 repetitions.

As shown in Figure 5, the external test results under the low-contamination condition (0.5 wt.%) revealed clear differences in the generalization ability of the evaluated models. Overall, PCA-SVM-RBF achieved the best performance, with Accuracy, Recall, F1, and AUC values of 0.996, 0.995, 0.997, and 1.000, respectively. To evaluate the statistical stability of the external-test performance, 95% confidence intervals were estimated by bootstrap resampling of the external test set with 1000 repetitions. The narrow confidence intervals of PCA-SVM-RBF indicate stable generalization performance under the low-contamination condition. These results indicate that this model could not only accurately distinguish clean and contaminated samples, but also maintain excellent stability and generalization capability on previously unseen external data. PLS-DA ranked second overall, with corresponding values of 0.980, 0.976, 0.982, and 0.988, suggesting that it was also highly suitable for this binary classification task and provided relatively balanced performance across all metrics. The predictive performance of PCA-LDA was slightly lower than that of PCA-SVM-RBF and PLS-DA, but it still maintained good classification ability, particularly in terms of Accuracy and F1. By contrast, although XGBoost achieved a relatively high AUC (0.993), indicating good overall separability between the two classes, its lower Recall and F1 suggest that some contaminated samples were still missed under the current decision setting. RF showed the weakest overall performance, with a particularly low Recall of only 0.692, indicating insufficient detection capability for contaminated samples and a tendency to misclassify them as clean powder. Overall, PCA-SVM-RBF demonstrated the best classification performance and generalization ability under the external test condition established in this study, followed by PLS-DA, whereas RF was not suitable as a preferred model for this task.

To further compare the proposed full-spectrum machine-learning models with a simple single-line baseline, a threshold-based classifier was constructed using the peak intensity of Si I 288.16 nm. The peak intensity was extracted from the ALS baseline-corrected spectra within a ±0.2 nm window around 288.16 nm, and the decision threshold was determined from the model-development dataset using the Youden index. The obtained threshold was then directly applied to the independent external test set. The results showed that the Si I 288.16 nm peak-intensity distributions of pure and contaminated samples strongly overlapped. On the external test set, the single-line threshold classifier achieved only 47.40% accuracy, 35.61% recall, 42.91% F1, and 0.479 AUC. The AUC values were 0.515 for the model-development dataset and 0.479 for the external test set, indicating that the discriminative ability of this single-line baseline was close to random classification. These results demonstrate that Si I 288.16 nm alone is insufficient for robust contamination identification under particle-flow LIBS conditions. In contrast, the full-spectrum machine-learning models achieved substantially better performance, further supporting the necessity of multivariate spectral classification rather than relying on a single emission line.

**Figure 6:**
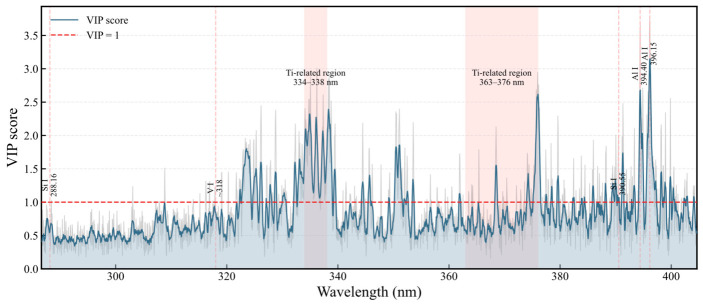
VIP-score profile of the full-spectrum PLS-DA model for the mixed-contamination binary classification task. The shaded regions indicate Ti-related spectral regions, and the dashed red line denotes the empirical VIP = 1 threshold. The gray line represents the wavelength-wise VIP-score fluctuations. High-VIP regions are mainly distributed around Ti-related matrix-emission bands at 334–338 nm and 363–376 nm, as well as Al/Si-related features around 390–396 nm.

To improve model interpretability, variable importance in projection (VIP) scores were calculated using the full-spectrum PLS-DA model for the mixed-contamination binary classification task. PLS-DA was selected because it directly uses the original wavelength variables and VIP analysis is widely applied in chemometric spectral interpretation. Variables with VIP scores greater than 1 were regarded as important contributors. As shown in Figure 6, the high-VIP regions were mainly distributed around Ti-related matrix-emission regions at 334–338 nm and 363–376 nm, together with the Al/Si-related region around 390–396 nm. These regions are consistent with the manually assigned characteristic lines in Section 3.1, indicating that the model captured chemically meaningful spectral variations. The result also suggests that contamination identification was not determined by a single characteristic peak, but by multivariate spectral redistribution involving both TC4 matrix-related features and AlSi10Mg-related features.

Although the above results suggest a relationship between model performance and contamination level, good performance in a single binary classification task does not necessarily prove that the model has learned the true spectral differences induced by contamination. It may also partially rely on factors that are incidentally correlated with the labels but are not intrinsically related to contamination, such as batch differences, overall intensity drift, baseline variation, instrument state, or sample-preparation fluctuations. In such cases, a model may achieve high apparent accuracy while depending on easily exploitable “shortcuts” rather than on genuinely transferable discriminative information [31]. If a model is only capable of binary classification, it may simply capture a coarse-grained difference. By contrast, if it can further distinguish among multiple adjacent contamination levels, it must rely on finer, more continuous, and more stable discriminative patterns rather than on a rough binary boundary. Therefore, a five-class classification experiment with different contamination levels was further conducted in this study to verify whether the spectral differences exhibited concentration-dependent fine-grained separability.

Table 2 summarizes the average confusion-matrix results of different models in the ten-fold cross-validation for the five-class task. Overall, the results confirm that the LIBS spectra corresponding to different contamination levels contain differences that can be learned and discriminated by the models, although the separability is not uniform across classes. The 5 wt.% class consistently exhibited the highest recognition rate across all models, indicating that a high contamination level induces more pronounced and stable spectral-response changes, thereby producing clearer class boundaries. In contrast, more obvious confusion was observed among the 0 wt.%, 0.5 wt.%, 1 wt.%, and 2 wt.% classes, with misclassifications mainly occurring between adjacent concentration levels. This suggests that the spectral response to contamination follows a more continuous transition rather than forming completely independent discrete patterns. In other words, within the low- and intermediate-contamination range, the contamination-induced spectral differences could already be captured by the models, but their magnitude remained close to the within-class variation, making class boundaries more likely to overlap.

A comparison between the training folds and the corresponding held-out folds further revealed differences in the generalization stability of the models. PCA-LDA and PCA-SVM-RBF achieved nearly 100% classification accuracy on the training folds while still maintaining relatively high accuracy on the held-out folds, indicating that these two models were able to extract stable discriminative features from the high-dimensional spectral data. In contrast, RF and XGBoost also achieved near-perfect classification on the training folds, but their accuracies decreased more noticeably on the held-out folds, reaching 86.13% and 92.28%, respectively. This indicates that tree-based ensemble models have a certain tendency to overfit high-dimensional single-shot LIBS spectra. Unlike PCA-based models, RF and XGBoost directly use the full wavelength variables and can capture highly localized spectral variations. Under particle-flow LIBS conditions, these local variations may originate not only from concentration-dependent compositional differences, but also from shot-to-shot plasma fluctuations, particle-position variations, and local peak-shape changes. Therefore, tree-based models may fit unstable spectral details in the training folds, leading to reduced generalization performance on held-out samples. The accuracies of PLS-DA on both the training and held-out folds were generally lower than those of PCA-LDA and PCA-SVM-RBF, indicating that its ability to characterize fine-grained concentration differences was relatively limited in the present multiclass problem. This limitation of PLS-DA was more evident in the class-wise results. PLS-DA showed relatively lower accuracy for some intermediate contamination levels, particularly for the 2 wt.% class. This can be attributed to the limitation of a linear latent-variable method when applied to nonlinear LIBS spectral responses. Under particle-flow LIBS conditions, the measured spectra are influenced not only by elemental composition, but also by matrix effects, shot-to-shot plasma fluctuations, particle-position variations, and local laser–particle coupling instability. These factors can cause non-monotonic changes in characteristic-line intensity and peak shape, leading to partial overlap among adjacent intermediate classes in the PLS-DA latent-variable space. In contrast, PCA-SVM-RBF can construct nonlinear decision boundaries after PCA-based dimensionality reduction, making it more suitable for handling overlapping and nonlinear spectral patterns. This result suggests that cross-contamination identification in flowing-powder LIBS should be treated as a multivariate and partially nonlinear classification problem rather than a purely linear concentration-response problem.

To examine whether regularization could reduce the overfitting tendency of tree-based models, regularized RF and XGBoost models were further evaluated. For RF, the maximum tree depth was limited, the minimum number of samples required for splitting and leaf nodes was increased, and square-root feature selection was used. The regularized RF model reduced the training accuracy to 97.76%, but the held-out accuracy remained similar to the original RF result, reaching 85.56%. This suggests that RF regularization reduced part of the training memorization ability, but did not substantially improve generalization for the present high-dimensional particle-flow LIBS spectra.

For XGBoost, stronger regularization was introduced by reducing the maximum tree depth, using subsampling and feature subsampling, increasing the minimum child weight, and adding L1 and L2 regularization terms. The regularized XGBoost model improved the held-out accuracy from 92.28% to 93.50%, and the training–held-out accuracy gap decreased to 6.50%. This indicates that regularization can moderately improve the robustness of XGBoost. Nevertheless, the remaining gap between training and held-out performance suggests that tree-based models are still sensitive to local spectral fluctuations. Overall, these results further support the use of PCA-SVM-RBF as the more robust model in this study, as PCA-based dimensionality reduction can suppress part of the high-dimensional spectral noise before nonlinear classification.

Finally, to examine the possibility of shortcut learning, a representative label-permutation control was conducted using the PCA-SVM-RBF model under the same five-class 10-fold cross-validation setting. The concentration labels were randomly shuffled while the spectral matrix remained unchanged. With the original labels, PCA-SVM-RBF achieved a 10-fold CV accuracy of 96.19% and a macro-F1 score of 96.18%. After label permutation, the accuracy decreased to 19.43%, and the macro-F1 score decreased to 19.39%, which are close to the random-chance level of 20% for the five-class task. This result suggests that the high classification performance was not caused by shortcut learning or random label associations, but reflected contamination-related spectral differences.

## 4. Conclusions

This study established an integrated LIBS-based measurement and classification framework for rapid identification of cross-contamination in metal powders for additive manufacturing. By combining stable particle-flow generation, cylindrical spatial confinement, single-shot spectral screening, and machine-learning classification, the proposed method enabled direct detection of flowing powder samples without pellet preparation. The results showed that the spectral differences induced by AlSi10Mg contamination in TC4 powder could be captured reliably, and the overall identification performance generally improved with increasing contamination level. Among the evaluated models, PCA-LDA and PLS-DA exhibited stable contamination-identification performance, whereas PCA-SVM-RBF achieved the best overall performance in low-concentration identification and external testing. The five-class results further demonstrated that the contamination-induced LIBS spectral response was concentration-dependent and followed a continuous transition pattern rather than a completely discrete distribution. Overall, the proposed method provides a feasible technical route for rapid identification of cross-contamination in additive-manufacturing metal powders and shows promising potential for online quality monitoring applications.

## Figures and Tables

**Table 1:** Hyperparameter settings of the machine-learning models.

Model	Binary Classification	Five-Class Classification
**PCA-LDA**	StandardScaler; PCA 95%; LDA default solver	Same as binary
**PLS-DA**	StandardScaler; 5 latent variables	StandardScaler; 4 latent variables
**PCA-SVM-RBF**	StandardScaler; PCA 95%; RBF kernel; C = 1.0; gamma = scale; probability = True	Same as binary; class_weight = balanced
**RF**	n_estimators = 300; max_depth = None; min_samples_split = 2; min_samples_leaf = 1; class_weight = balanced	n_estimators = 400; max_depth = None; min_samples_split = 2; min_samples_leaf = 1; class_weight = balanced
**XGBoost**	n_estimators = 300; max_depth = 4; learning_rate = 0.05; subsample = 0.9; colsample_bytree = 0.9; objective = binary:logistic; eval_metric = logloss	n_estimators = 400; max_depth = 5; learning_rate = 0.05; subsample = 0.9; colsample_bytree = 0.9; objective = multi:softprob; eval_metric = mlogloss

**Table 2:** Average ten-fold cross-validation confusion matrices for the five contamination levels.

PCA-LDA													
Prediction results of cross-validation training folds							Prediction results of cross-validation hold-out folds						
	0.5%	0%	1%	2%	5%	Accuracy		0.5%	0%	1%	2%	5%	Accuracy
0.5%	881.0	0.1	0.0	0.0	0.0	99.99%	0.5%	94.8	1.1	1.4	0.2	0.4	96.83%
0%	1.9	886.8	0.1	0.0	0.4	99.73%	0%	1.3	95.3	2.1	0.0	0.1	96.46%
1%	0.4	0.0	914.0	0.0	0.0	99.96%	1%	1.3	1.6	98.4	0.2	0.1	96.85%
2%	0.3	0.0	1.0	832.0	0.1	99.83%	2%	1.7	0.8	1.7	88.0	0.4	95.03%
5%	0.0	0.0	0.0	0.0	900.0	100.00%	5%	0.0	0.0	0.0	0.0	100.0	100.00%
Overall accuracy in CV training folds						99.90%	Overall accuracy in cross-validation hold-out folds						97.07%
PLS-DA													
Prediction results of cross-validation training folds							Prediction results of cross-validation hold-out folds						
	0.5%	0%	1%	2%	5%	Accuracy		0.5%	0%	1%	2%	5%	Accuracy
0.5%	689.1	91.2	30.9	4.5	65.4	78.21%	0.5%	75.7	10.5	3.6	0.5	7.6	77.32%
0%	33.9	810.0	36.7	1.7	6.9	91.09%	0%	4.0	88.9	4.8	0.3	0.8	89.98%
1%	89.3	56.2	749.8	0.0	19.1	82.00%	1%	10.9	6.6	81.7	0.0	2.4	80.41%
2%	113.5	38.7	72.9	569.5	38.8	68.33%	2%	12.2	4.3	8.7	62.9	4.5	67.93%
5%	0.0	0.0	4.1	0.0	895.9	99.54%	5%	0.0	0.0	0.5	0.0	99.5	99.50%
Overall accuracy in CV training folds						84.07%	Overall accuracy in cross-validation hold-out folds						83.26%
PCA-SVM-RBF													
Prediction results of cross-validation training folds							Prediction results of cross-validation hold-out folds						
	0.5%	0%	1%	2%	5%	Accuracy		0.5%	0%	1%	2%	5%	Accuracy
0.5%	880.8	0.0	0.3	0.0	0.0	99.97%	0.5%	94.0	1.9	1.7	0.2	0.1	96.02%
0%	1.0	888.1	0.1	0.0	0.0	99.88%	0%	1.4	94.8	2.3	0.3	0.0	95.95%
1%	1.0	0.0	913.4	0.0	0.0	99.89%	1%	2.6	1.0	98.0	0.0	0.0	96.46%
2%	0.3	0.0	0.0	833.1	0.0	99.96%	2%	2.1	1.1	2.9	85.8	0.7	92.66%
5%	0.0	0.0	1.2	0.0	898.8	99.87%	5%	0.0	0.0	0.0	0.4	99.6	99.60%
Overall accuracy in CV training folds						99.91%	Overall accuracy in cross-validation hold-out folds						96.19%
RF													
Prediction results of cross-validation training folds							Prediction results of cross-validation hold-out folds						
	0.5%	0%	1%	2%	5%	Accuracy		0.5%	0%	1%	2%	5%	Accuracy
0.5%	881.1	0.0	0.0	0.0	0.0	100.00%	0.5%	81.7	8.4	4.3	2.5	1.0	83.45%
0%	0.0	889.2	0.0	0.0	0.0	100.00%	0%	4.4	85.4	7.2	1.5	0.3	86.44%
1%	0.0	0.0	914.4	0.0	0.0	100.00%	1%	10.6	8.0	78.7	3.7	0.6	77.46%
2%	0.0	0.0	0.0	833.4	0.0	100.00%	2%	4.0	1.8	7.2	78.0	1.6	84.23%
5%	0.0	0.0	0.0	0.0	900.0	100.00%	5%	0.0	0.1	0.9	0.0	99.0	99.00%
Overall accuracy in CV training folds						100.00%	Overall accuracy in cross-validation hold-out folds						86.13%
XGBoost													
Prediction results of cross-validation training folds							Prediction results of cross-validation hold-out folds						
	0.5%	0%	1%	2%	5%	Accuracy		0.5%	0%	1%	2%	5%	Accuracy
0.5%	881.1	0.0	0.0	0.0	0.0	100.00%	0.5%	87.0	4.9	3.9	1.5	0.6	88.87%
0%	0.0	889.2	0.0	0.0	0.0	100.00%	0%	4.5	90.3	3.5	0.2	0.3	91.40%
1%	0.0	0.0	914.4	0.0	0.0	100.00%	1%	4.3	4.8	90.5	1.5	0.5	89.07%
2%	0.0	0.0	0.0	833.4	0.0	100.00%	2%	1.6	1.1	3.4	85.7	0.8	92.55%
5%	0.0	0.0	0.0	0.0	900.0	100.00%	5%	0.1	0.0	0.4	0.0	99.5	99.50%
Overall accuracy in CV training folds						100.00%	Overall accuracy in cross-validation hold-out folds						92.28%

## Data Availability

Data will be made available on request.

## Abbreviations

The following abbreviations are used in this manuscript:

LIBS	Laser-Induced Breakdown Spectroscopy
LDA	Linear Discriminant Analysis
PLS-DA	Partial Least Squares Discriminant Analysis
SVM-RBF	Support Vector Machine with Radial Basis Function kernel
RF	Random Forest
XGBoost	Extreme Gradient Boosting
